# An Improved Classification Method Using a New Generalized Base Belief Function

**DOI:** 10.3390/e28101098

**Published:** 2026-10-09

**Authors:** Qi Wei, Shiqi Qiang, Hangjun Li, Shan He, Junqiang Liu, Longxiang Hou, Yongchuan Tang

**Affiliations:** 1 School of Integrated Circuits and Microelectronics, Northwestern Polytechnical University, Xi’an 710072, China; 2 Shenzhen Research Institute of Northwestern Polytechnical University, Shenzhen 518057, China; 3 School of Automation, Northwestern Polytechnical University, Xi’an 710072, China

**Keywords:** Dempster–Shafer evidence theory, open-world classification, generalized base belief function, evidence modification, conflict data fusion

## Abstract

Open-world classification must account for samples whose classes are absent from the current frame of discernment. Although generalized evidence theory can represent such possibilities, the conventional base belief function assigns reference support only to nonempty propositions and therefore cannot directly support source-level treatment of unknown information. This work proposes a generalized base belief function that incorporates the open-world proposition into its reference domain and uses it to modify generalized basic probability assignments before fusion. The modified evidence is combined through rules compatible with generalized evidence theory and followed by a known/unknown decision, forming a complete classification procedure for incomplete frames. Experiments on Iris and Seeds show that source-level modification and generalized fusion jointly support known/unknown classification. On Waveform, classification remains stable as controlled perturbation increases in a larger, higher-dimensional, noisy environment. On the Wall-Following Robot dataset, the procedure gives a selective known/unknown decision across different class compositions, preserving known samples while retaining overall discrimination close to GCR. These results support the proposed GBBF-based method for open-world classification under incomplete frames.

## 1. Introduction

Dempster–Shafer evidence theory (D-S theory) [1,2] provides a flexible framework for modeling and processing uncertain information [3]. It has been applied to target recognition [4], classification [5,6,7], clustering [8,9], decision making [10], sensor data fusion [11], and related uncertainty-processing tasks [12,13,14,15]. In classification, however, the available frame of discernment may be incomplete because a test sample can originate from a class that was not modeled when the frame was constructed [16]. Under this open-world assumption, generalized evidence theory (GET) permits m(∅)>0, so that ∅ explicitly represents possibilities outside the current frame. This representation provides the basis for distinguishing known classes from alternatives outside the frame, and data-driven mass construction has also been developed for open-world fusion and classification [17].

Conflict management can proceed at two levels. Rule-level methods redistribute or normalize conflict during combination [18,19], whereas source-level methods adjust evidence reliability or representation before fusion [20,21,22,23,24]. Both depend on the assumptions made about the information sources [25,26]. For incomplete frames, the transferable belief model [27] and GET [28] allow uncertainty outside the specified frame to be represented through the empty set, while mGCR adapts evidence combination to this setting [29,30]. At the source level, conventional base belief functions provide reference distributions for evidence modification [31,32], but they are constructed only over nonempty propositions. Consequently, when ∅ represents possibilities outside an incomplete frame, a conventional BBF cannot directly provide reference support for this open-world proposition. Gan et al. [33] incorporated frame size and nonzero empty-set mass into an extended BBF for non-exhaustive frames. The GBBF developed here instead defines a fixed maximum-entropy uniform reference over the complete generalized proposition space, including ∅, and uses it for α-parameterized source-level GBPA modification. This reference is integrated with evidence-condition assessment and open-world classification, so that incomplete-frame uncertainty enters the evidence representation before fusion.

The main contribution of this work is an improved open-world classification method built around the proposed GBBF. The procedure links GBBF-based evidence modification with evidence-condition assessment, GET-compatible fusion, and a known/unknown decision. The mathematical properties of the GBBF are analyzed theoretically. The experiments first examine the source-modification and fusion mechanisms on Iris and Seeds. Waveform is then used to evaluate classification stability in a substantially higher-dimensional noisy setting, while Wall-Following Robot tests the method under different known/unknown class compositions.

The remainder of this paper is organized as follows. Section 2 reviews the necessary preliminaries. Section 3 introduces the generalized base belief function and the evidence-modification mechanism under the open-world assumption. Section 4 presents the corresponding improved classification method. Section 5 reports the experimental design, mechanism-oriented validation, stability analysis, structural generalization, and discussion. Section 6 concludes the paper.

## 2. Preliminaries

### 2.1. Dempster–Shafer Evidence Theory

Let the frame of discernment (FOD) Ω contain *N* mutually exclusive hypotheses:

(1) $$\Omega =\{H_{1},H_{2},\ldots ,H_{N}\}$$

Its power set contains $2^{N}$ propositions:

(2) $$2^{\Omega }=\left\{\emptyset ,\left\{H_{1}\right\},\left\{H_{2}\right\},\ldots ,\left\{H_{N}\right\},\left\{H_{1}\cup H_{2}\right\},\ldots ,\Omega \right\}$$

A basic probability assignment (BPA), also called a mass function, is a mapping $m:2^{\Omega }\to \left[0,1\right]$ satisfying

(3) m(∅)=0

(4) $$\sum_{A\in 2^{\Omega }}m(A)=1$$

Two independent BPAs $m_{1}$ and $m_{2}$ are fused by Dempster’s combination rule [1]:

(5) $$m\left(A\right)=\left(m_{1}⊕m_{2}\right)\left(A\right)\;=\frac{1}{1-k}\sum_{B\cap C=A}m_{1}\left(B\right)m_{2}\left(C\right),$$

where the conflict coefficient *k* is

(6) $$k=\sum_{B\cap C=\emptyset }m_{1}\left(B\right)m_{2}\left(C\right).$$

Here, k=0 indicates no pairwise conflict and k=1 complete conflict. These definitions provide the closed-frame notation from which GET relaxes the restriction m(∅)=0.

### 2.2. Improved Combination Rules Under the Open-World Assumption

Under the open-world assumption, generalized evidence theory (GET) extends the BPA to a generalized basic probability assignment (GBPA) and Dempster’s rule to the generalized combination rule (GCR) [28]. Suppose that *U* is a frame of discernment under the open-world assumption with a power set $2^{U}$. For each $A\in 2^{U}$, a GBPA is a mapping $m:2^{U}\to \left[0,1\right]$ that satisfies [28]:

(7) $$\sum_{A\in 2^{U}}m(A)=1$$

If m(∅)=0, the GBPA reduces to a conventional BPA.

In GET, ∅ represents the open-world proposition. GCR assigns masses to the ordinary propositions according to [28]

(8) $$m\left(A\right)=\frac{\left(1-m(\emptyset )\right)}{1-K}\sum_{B\cap C=A}m_{1}\left(B\right)m_{2}\left(C\right),$$

where $K=\sum_{B\cap C=\emptyset }m_{1}\left(B\right)m_{2}\left(C\right)$ and $m\left(\emptyset \right)=m_{1}\left(\emptyset \right)m_{2}\left(\emptyset \right)$; the limiting case K=1 is treated as specified in [28].

The modified GCR (mGCR) evaluates the ordinary-proposition masses according to [29]

(9) $$m\left(A\right)=\frac{\sum_{B\cap C=A}m_{1}\left(B\right)m_{2}\left(C\right)}{1-K},$$

(10) $$m\left(\emptyset \right)=\frac{m_{1}\left(\emptyset \right)m_{2}\left(\emptyset \right)}{1-K},$$

where $K=\sum_{\begin{array}{c} B\cap C=\emptyset \\ B\cup C\neq \emptyset \end{array}}m_{1}\left(B\right)m_{2}\left(C\right)$. If K=1, or if all nonempty propositions have zero mass, m(∅)=1. If both input GBPAs assign zero mass to ∅, mGCR reduces to Dempster’s combination rule. The GBPA representation and these GET-compatible combination rules form the basis for the source-modification mechanism introduced next.

## 3. Generalized Base Belief Function Under Incomplete Information

### 3.1. Generalized Base Belief Function

Under the open-world assumption, $U=\{H_{1},H_{2},\ldots ,H_{N}\}$ contains *N* mutually exclusive hypotheses, and $2^{U}$ contains $2^{N}$ propositions. Since a nonzero m(∅) supports possibilities outside *U*, a reference function for source modification should include the open-world proposition while remaining normalized. Accordingly, we define the generalized base belief function $m_{b}$ on every proposition $A\in 2^{U}$ as

(11) $$m_{b}\left(A\right)=\frac{1}{2^{N}},\;A\in 2^{U}.$$

The conventional base belief function [31,32] assigns $m_{\mathrm{BBF}}\left(A\right)=1/\left(2^{N}-1\right)$ for A≠∅ and $m_{\mathrm{BBF}}\left(\emptyset \right)=0$, thereby excluding ∅ from its reference domain. The GBBF instead covers every proposition in $2^{U}$. Under normalization, the uniform assignment uniquely maximizes $-\sum_{A\in 2^{U}}q\left(A\right)\log q\left(A\right)$ and thus attains the maximum Shannon entropy. The uniform GBBF serves as an epistemically neutral reference; proposition-specific prior information can instead be represented by a normalized nonuniform *q*. For the uniform GBBF considered here, the modification strength is parameterized by α as follows:

(12) $$m_{\alpha }\left(A\right)=\left(1-\alpha \right)m\left(A\right)+\alpha m_{b}\left(A\right),\;0\leq \alpha \leq 1.$$

The numerical examples and the Iris and Seeds experiments use the symmetric setting α=0.5, corresponding to equal weighting of the original GBPA and the GBBF reference. For the cross-validated extended evaluations, application-dependent parameters are determined from the training data and fixed before testing. Under the symmetric setting,

(13) $$m^{\prime }\left(A\right)=\frac{m_{b}\left(A\right)+m\left(A\right)}{2}.$$

Here, *m* retains the source-specific mass structure, $m_{b}$ supplies the neutral reference, and α controls the modification strength. Convexity preserves nonnegativity and normalization, $\sum_{A\in 2^{U}}m_{\alpha }\left(A\right)=1$, with $m_{0}=m$ and $m_{1}=m_{b}$. Thus, Equation (12) interpolates between the original GBPA and the GBBF. Even if m(∅)=0, any α>0 gives $m_{\alpha }\left(\emptyset \right)=\alpha 2^{-N}>0$, so the open-world component is present in the modified source before combination. Hereafter, a GBPA modified by the GBBF is denoted as mGBPA.

As *N* increases, the per-proposition reference contribution $\alpha 2^{-N}$ decreases while the total mixing weight remains controlled by α. The resulting influence depends on the focal-set structure and subsequent fusion. Explicit construction of the complete proposition space scales exponentially with *N*, which motivates efficient representations for large frames. For a frame with *N* elements, the complete generalized proposition space contains $2^{N}$ subsets; a direct dense implementation therefore requires $O(2^{N})$ storage and $O(2^{N})$ operations for source-level GBBF modification. If two dense mass functions are combined by naive pairwise enumeration, the number of proposition pairs can reach $O(4^{N})$ in the worst case. This motivates sparse focal-set representations, pruning, or approximate fusion for larger frames.

### 3.2. Numerical Examples

The following examples examine the effect of the proposed modification under two distinct open-world evidence configurations.

**Example** **1.** *This example examines a low-conflict case with no initial support for the open-world proposition. Define the frame of discernment U={a,b} under the open-world assumption; two GBPAs are given as follows:*

$$m_{1}\left(a\right)=0.98,m_{1}\left(b\right)=0.02,\;m_{2}\left(a\right)=0.98,m_{2}\left(b\right)=0.02.$$

The generalized base belief function for the numerical example is given by:

$$m_{b}\left(a\right)=m_{b}\left(b\right)=m_{b}\left(ab\right)=m_{b}\left(\emptyset \right)=\frac{1}{4}.$$

The two GBPAs after modification based on Equation (13) are as follows:

$${m_{1}}^{\prime }\left(a\right)=\frac{\frac{1}{4}+m_{1}\left(a\right)}{2}=0.615,{m_{1}}^{\prime }\left(b\right)=\frac{\frac{1}{4}+m_{1}\left(b\right)}{2}=0.135,\;{m_{1}}^{\prime }\left(a,b\right)=\frac{\frac{1}{4}+m_{1}\left(a,b\right)}{2}=0.125,{m_{1}}^{\prime }\left(\emptyset \right)=\frac{\frac{1}{4}+m_{1}\left(\emptyset \right)}{2}=0.125.$$

$${m_{2}}^{\prime }\left(a\right)=\frac{\frac{1}{4}+m_{2}\left(a\right)}{2}=0.615,{m_{2}}^{\prime }\left(b\right)=\frac{\frac{1}{4}+m_{2}\left(b\right)}{2}=0.135,\;{m_{2}}^{\prime }\left(a,b\right)=\frac{\frac{1}{4}+m_{2}\left(a,b\right)}{2}=0.125,{m_{2}}^{\prime }\left(\emptyset \right)=\frac{\frac{1}{4}+m_{2}\left(\emptyset \right)}{2}=0.125.$$

The combination result of the modified GBPAs with mGCR is as follows:

m(a)=0.8647,m(b)=0.0845,m(a,b)=0.0254,m(∅)=0.0254.

Neither original GBPA assigns mass to ∅. After GBBF-based modification, both sources provide explicit open-world reference support with $m_{1}^{\prime }\left(\emptyset \right)=m_{2}^{\prime }\left(\emptyset \right)=0.125$. After mGCR fusion, *a* remains dominant with m(a)=0.8647, while m(∅)=0.0254. The modification therefore introduces an open-world component without disrupting the dominant support structure of the original evidence.

**Example** **2.** *This example examines stronger support for the open-world proposition and compares the available combination strategies. Define the frame of discernment U={a,b,c} under the open-world assumption; two GBPAs are given as follows:*

$$m_{1}\left(a\right)=0.2,m_{1}\left(b\right)=0.2,m_{1}\left(\emptyset \right)=0.6\;m_{2}\left(a\right)=0.1,m_{2}\left(b,c\right)=0.1,m_{2}\left(\emptyset \right)=0.8$$

The fusion results obtained with the four available strategies are shown in Table 1. The fused mass assigned to ∅ by mGBPA&mGCR is 0.4156.

The stronger initial support for ∅ separates the two mechanisms: GBBF modification redistributes source-level support across the generalized proposition space, whereas mGCR retains more empty-set support during fusion than GCR. They are therefore complementary rather than redundant.

## 4. Improved Classification Method Based on the Generalized Base Belief Function

Based on the GBBF mechanism introduced above, the proposed classification method integrates source-level GBPA modification with evidence-condition assessment, GET-compatible fusion, and a known/unknown decision. The procedure is implemented in four steps: generate GBPAs, assess the evidence condition, fuse the evidence, and assign a known or unknown class label.

Step 1. Generate GBPAs.

Under the open-world assumption, GBPAs are generated for each attribute using the triangular fuzzy-number model in [34].

Step 2. Assess the evidence condition.

The evidence condition determines which combination strategy is used. For evidence sources *i* and *j*, the routing conflict coefficient is defined using the mGCR-compatible pairwise form

(14) $$K_{ij}=\sum_{\begin{array}{c} B\cap C=\emptyset \\ B\cup C\neq \emptyset \end{array}}m_{i}\left(B\right)m_{j}\left(C\right),\;K_{\max}=\max_{i<j}K_{ij}.$$

The mutually supportive empty–empty product is excluded from this pairwise conflict, whereas the *K* in GCR includes all empty intersections. Here, $K_{\max}$ measures the strongest pairwise incompatibility, while $s_{\emptyset }=\max_{l}m_{l}\left(\emptyset \right)$ measures the strongest direct open-world support, where *l* indexes the evidence sources. Together they determine the fusion strategy. Thresholds $\tau_{K}$ and $\tau_{\emptyset }$ are application-dependent routing parameters used to separate the three evidence conditions. For the cross-validated experiments, application-dependent routing parameters and, where applicable, the modification strength are determined from training-side information and fixed before evaluation of the corresponding test fold.

- Condition 1: $K_{\max}<\tau_{K}$ and $s_{\emptyset }<\tau_{\emptyset }$; data fusion has no substantial conflict and support for ∅ is weak.
- Condition 2: $K_{\max}<\tau_{K}$ and $s_{\emptyset }\geq \tau_{\emptyset }$; data fusion has no substantial conflict and support for ∅ is strong.
- Condition 3: $K_{\max}\geq \tau_{K}$; data fusion has substantial conflict.

Step 3. Evidence fusion.

For Condition 1, GCR is used directly. For Condition 2, mGCR is used to retain the role of the open-world proposition. For Condition 3, each GBPA is first modified using the generalized base belief function and the resulting mGBPAs are fused by mGCR.

Step 4. Classification decision.

Let $m_{F}$ denote the fused GBPA. The final decision selects the candidate with the largest fused mass among the known-class singletons and the open-world proposition:

(15) $$\hat{y}=\arg\max_{A\in \{\left\{H_{1}\right\},\ldots ,\left\{H_{N}\right\},\emptyset \}}m_{F}\left(A\right).$$

A maximizing singleton denotes the corresponding known class, whereas a maximizing ∅ denotes an unknown class. Composite propositions represent residual imprecision and are not treated as direct class labels.

**Figure 1:**
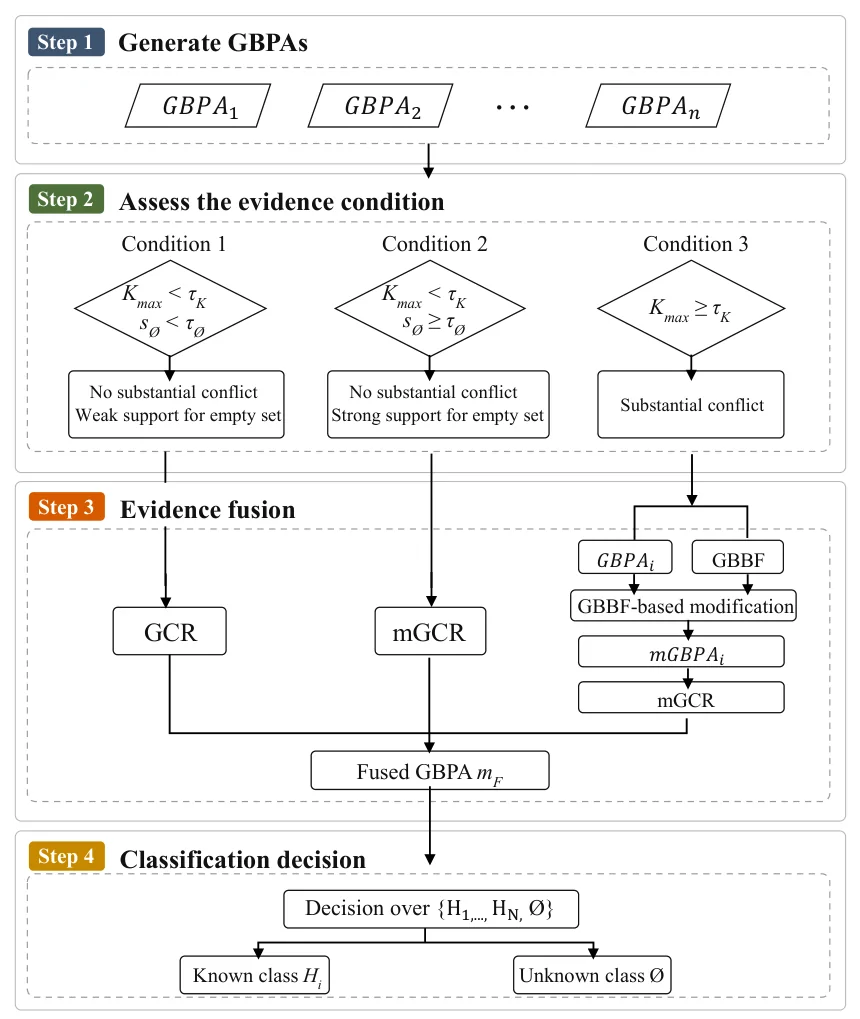
Workflow of the proposed GBBF-based classification method.

The complete classification procedure is summarized in Figure 1.

## 5. Experimental Evaluation and Analysis

### 5.1. Experimental Design and Evaluation Metrics

The experiments proceed from interpretable low-dimensional settings on Iris and Seeds to cross-validated evaluation on the larger Waveform and Wall-Following Robot datasets. Iris and Seeds illustrate GBPA construction, GBBF modification, fusion behavior, and the resulting known/unknown decision. The larger datasets introduce more attributes as evidence sources and broader open-world configurations. The characteristics of the four datasets are summarized in Table 2.

For compact notation, *x*K/*y*U denotes an open-world setting with *x* known classes and *y* withheld classes treated as unknown.

The Iris and Seeds experiments use their established fixed training/test splits and report representative evidence together with full-test-set classification. The extended evaluations cover all three 2K/1U rotations for Waveform, all four 3K/1U rotations for Robot, and all six 2K/2U Robot configurations. Each configuration is evaluated with five outer folds: Waveform uses stratified folds, whereas the sequential Robot observations are divided into five contiguous source-order blocks.

For each outer fold, the class-balanced training budget and α are selected exclusively from the corresponding outer-training data by three-fold inner validation with pseudo-unknown classes and three deterministic resamples. The routing thresholds $\tau_{K}$ and $\tau_{\emptyset }$ are then calibrated from the same training data. All parameter selection is completed within the outer-training data before evaluation of the held-out fold. Results are reported as mean ± sample standard deviation across the outer-test-fold units: 15 for Waveform, 20 for Robot 3K/1U, and 30 for Robot 2K/2U.

The same GBPA-generation procedure is retained across all comparative configurations, so differences among the configurations arise from the subsequent source-modification and fusion strategies rather than from GBPA generation.

Accuracy and macro-F1 measure overall and class-balanced classification performance. The unknown-detection rate is $\mathrm{UDR}=n_{\mathrm{unknown}\to \emptyset }/n_{\mathrm{unknown}}$, and the false-rejection rate is $\mathrm{FRR}=n_{\mathrm{known}\to \emptyset }/n_{\mathrm{known}}$. All reported mass vectors are presented to four decimal places while preserving the normalization condition. The datasets are obtained from the UCI Machine Learning Repository [35,36,37,38].

To characterize the known/unknown decision across operating points, we use the final fused-mass score

(16) $$s\left(x\right)=m_{F}\left(\emptyset \right)-\max_{i}m_{F}\left(\left\{H_{i}\right\}\right).$$

Its sign expresses the empty-set versus singleton comparison in Equation (15), with the existing tie rule retained under the original argmax decision rule. Sweeping an evaluation threshold *t* in s(x)≥t gives the complete UDR–FRR trade-off without changing the trained model or its evidence-condition parameters. AUROC summarizes this threshold-independent discrimination, taking unknown samples as positive. For Robot, the five held-out folds are combined within each known/unknown configuration before the configuration curves are averaged on a common FRR grid.

### 5.2. Classification Evaluation on Iris and Seeds

#### 5.2.1. Known-Class Classification on the Iris Dataset

**Figure 2:**
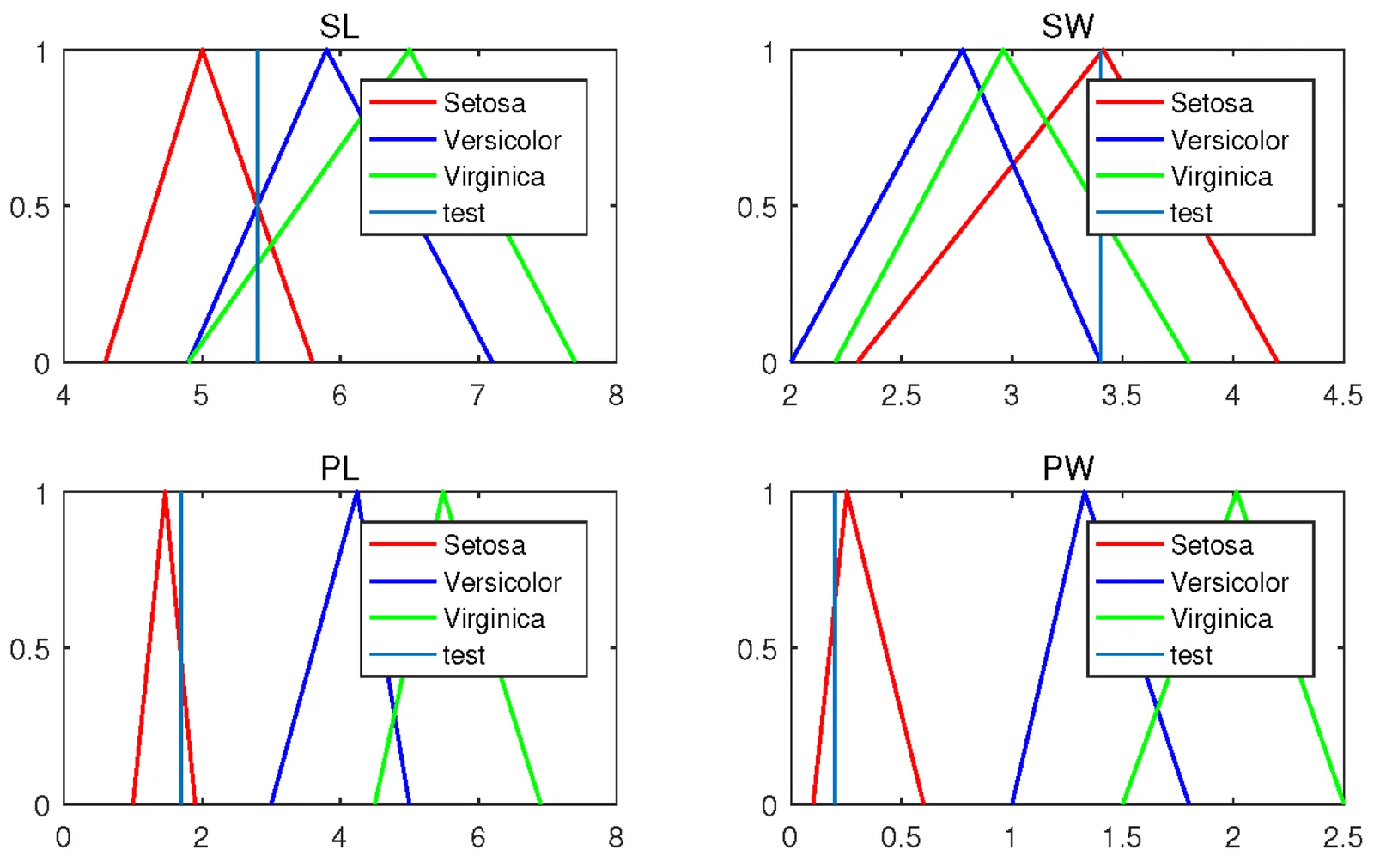
Triangular fuzzy-number models for the Iris known-class setting.

The first experiment examines known-class discrimination before an unknown class is introduced. The three categories of the UCI Iris dataset are Setosa (a), Versicolor (b), and Virginica (c). Each class contains 50 Iris instances described by sepal length (SL), sepal width (SW), petal length (PL), and petal width (PW). For the fixed split, 30 instances from each class are used for training and the remaining 20 instances are used for testing. All test samples therefore belong to known classes, and the GBPA representation continues to include ∅ as the open-world proposition. The GBPA-generation method in [34] yields the triangular fuzzy-number models shown in Figure 2.

For the representative Setosa (a) instance (5.4, 3.4, 1.7, 0.2), the resulting attribute-level GBPAs are given in Table 3.

Table 4 compares the four fusion strategies for the representative known-class instance. Each assigns its largest mass to Setosa (a), while the distributions over the remaining propositions and ∅ differ.

The four attribute-level sources are recursively fused in the order SL, SW, PL, and PW. For the full known-class test set, the original GBPAs are fused by mGCR without GBBF modification. The correct-classification rates are 100% for Setosa, 90% for Versicolor, and 85% for Virginica, giving an overall accuracy of 91.67%. The underlying GBPA construction and fusion process therefore retain effective discrimination among the known classes before the open-world experiments are introduced.

#### 5.2.2. Open-World Classification on the Iris Dataset

**Figure 3:**
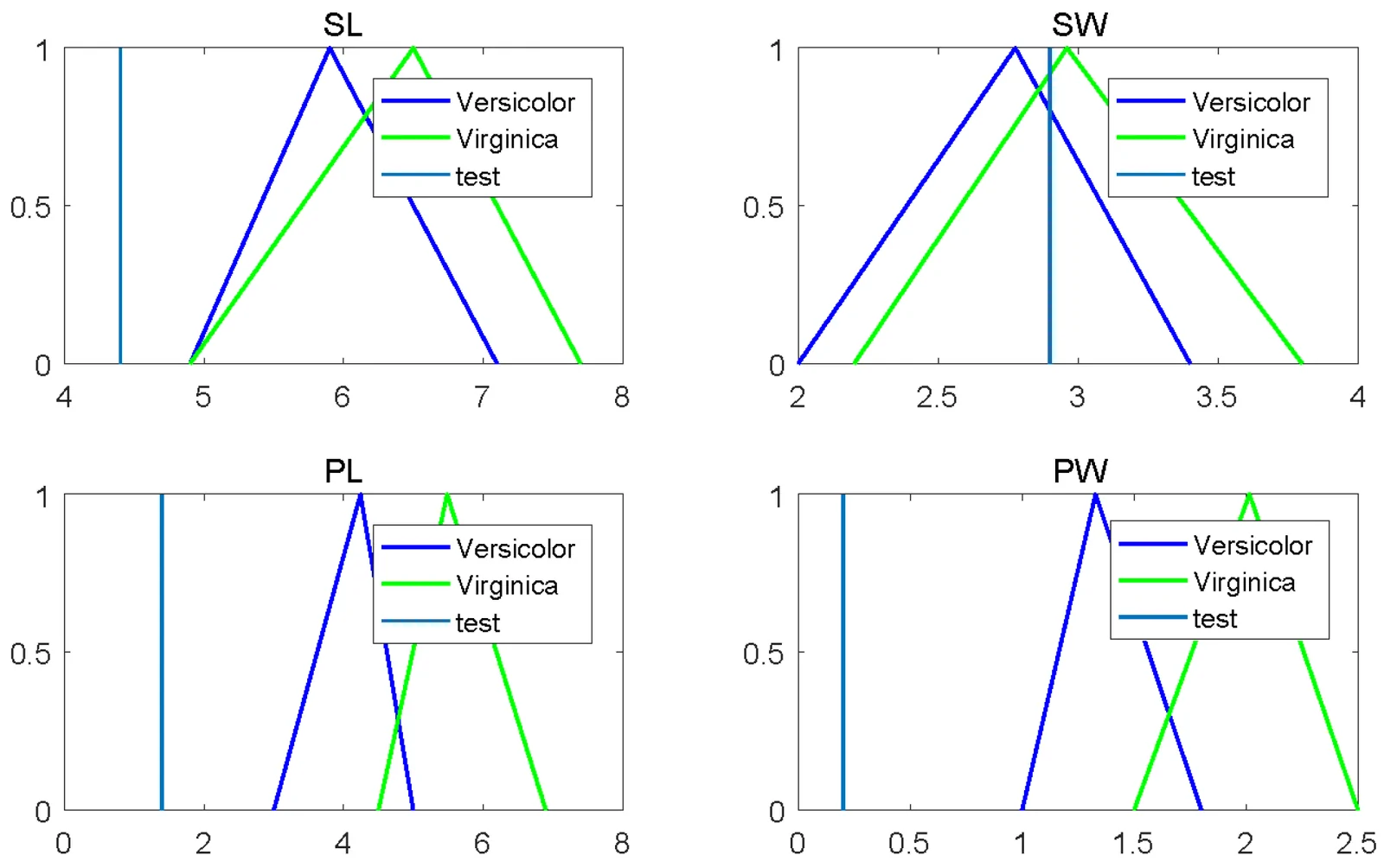
Triangular fuzzy-number models for the Iris open-world setting.

The next experiment evaluates an open-world Iris setting in which only two of the three Iris classes are available during training and the withheld class is treated as unknown during testing. For the fixed split, 30 Versicolor (b) and 30 Virginica (c) samples are selected for training. The remaining 20 samples from each known class and all 50 Setosa (a) samples are used for testing, with Setosa treated as an unknown class. The triangular fuzzy-number models generated using [34] are shown in Figure 3.

For the representative Setosa (a) instance (4.4, 2.9, 1.4, 0.2), Table 5 reports the attribute-level GBPAs before and after GBBF-based evidence modification.

To isolate the effect of the combination rule, Table 6 fuses the same GBBF-modified evidence using GCR and mGCR, respectively.

For this representative instance, mGBPA&GCR assigns its largest fused mass to Virginica (c), whereas mGBPA&mGCR retains substantially more open-world support and makes m(∅)=0.6650 the largest mass. Under the decision rule in Section 4, the withheld Setosa sample is therefore identified as unknown through the empty-set proposition. The comparison separates two complementary roles: GBBF introduces open-world support at the source level, while mGCR preserves more of this support during fusion. This behavior supports the correct identification of evidence originating outside the current frame.

The representative result is complemented by an evaluation over the full set of 90 test samples. The confusion matrix is given in Table 7; rows denote predicted labels and columns denote actual labels.

The test-set accuracy is 86/90=95.56%, with Cohen’s κ=0.9245 and macro-F1 of 0.9377. All 50 withheld Setosa samples are detected as unknown, giving an unknown-detection rate of 50/50=100%. Only one of the 40 known-class samples is rejected, corresponding to a false-rejection rate of 1/40=2.5%. These results show that the proposed method identifies samples outside the known-class set through the open-world proposition while largely preserving the classification of the known samples in this Iris setting.

#### 5.2.3. Open-World Classification on the Seeds Dataset

**Figure 4:**
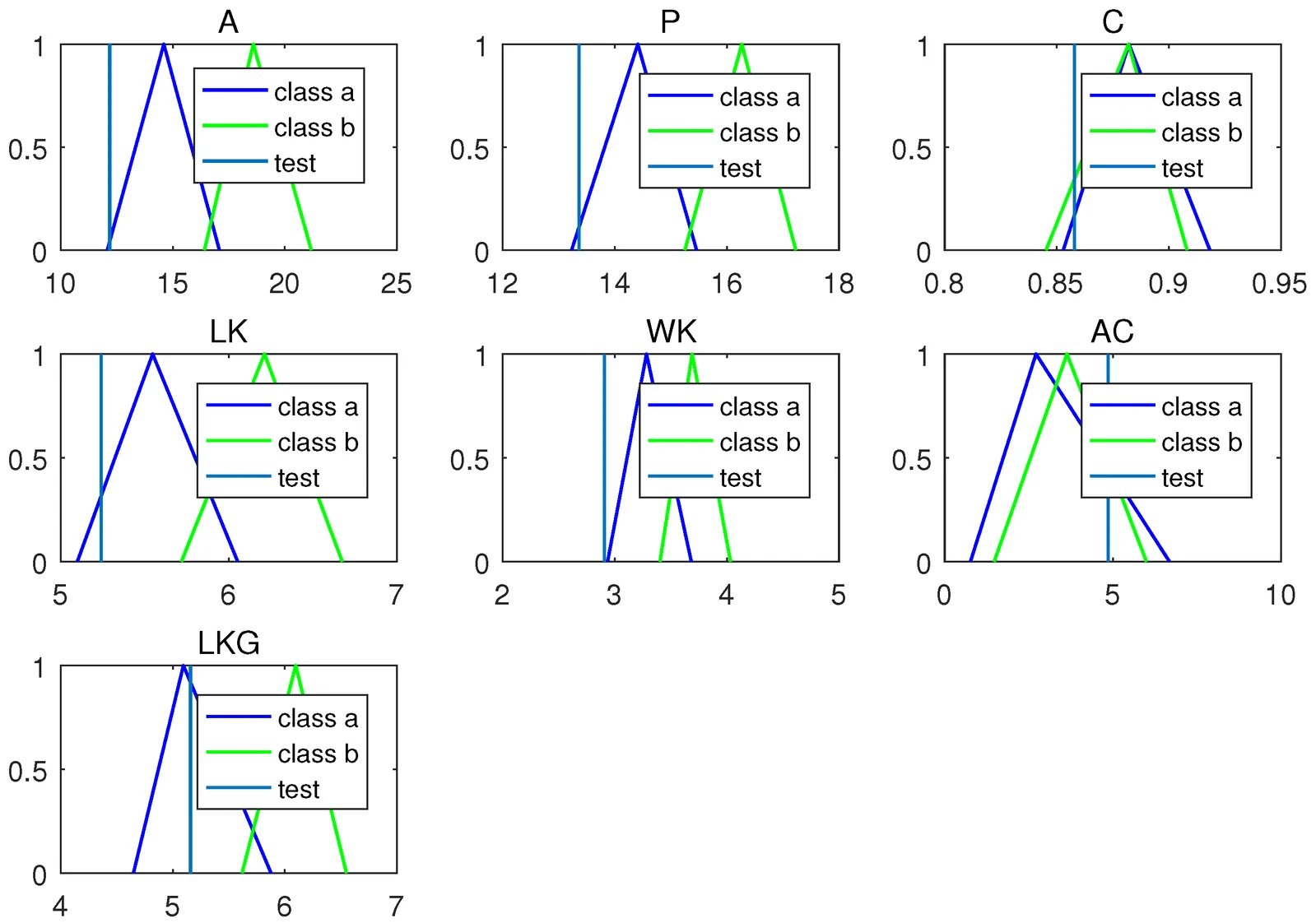
Triangular fuzzy-number models for the Seeds open-world setting.

Seeds provides a further open-world test in a different feature space and class distribution. Forty samples from each of classes *a* and *b* are selected for training, class *c* is treated as unknown, and the test set contains the remaining 30 samples from each known class together with all 70 samples from class *c*. The same GBPA-generation procedure and GBBF-modification–mGCR strategy are used to examine the open-world evidence mechanism in this second feature space. The seven attributes are area (A), perimeter (P), compactness (C), length of kernel (LK), width of kernel (WK), asymmetry coefficient (AC), and length of kernel groove (LKG) [36]. The corresponding triangular fuzzy-number models are shown in Figure 4.

A representative sample from the withheld class *c* has the seven-attribute vector

(12.19,13.36,0.8579,5.24,2.909,4.857,5.158).

Table 8 reports its original and GBBF-modified attribute-level GBPAs.

Using the same GBBF-modified evidence, Table 9 compares the corresponding GCR and mGCR fusion results.

A similar contrast is observed for the Seeds instance: mGBPA&GCR favors known class *a*, whereas mGBPA&mGCR retains sufficient open-world support for the unknown class to become dominant. The representative class-*c* sample is therefore correctly identified as unknown.

The resulting test-set confusion matrix is shown in Table 10; the two known classes are denoted by *a* and *b*, while the withheld class is represented by ∅ in the decision.

The test-set accuracy is 113/130=86.92%, with Cohen’s κ=0.7923 and macro-F1 of 0.8584. Of the 70 withheld class-*c* samples, 59 are detected as unknown and 11 are assigned to class *a*, giving an unknown-detection rate of 59/70=84.29%. None of the 60 known-class samples is rejected, so the false-rejection rate is 0/60=0. The proposed method therefore identifies most withheld Seeds samples as unknown while preserving all known samples.

### 5.3. Classification Stability on High-Dimensional Noisy Data

The preceding experiments use four or seven attributes to make the evidence mechanism directly interpretable. The Waveform Database Generator Version 1 extends the evaluation to 5000 samples, 21 continuous attributes, and three classes [37]. Each attribute acts as an evidence source. The dataset combines overlapping waveform classes with independent additive noise in every attribute, providing a higher-dimensional noisy setting for evaluating classification stability.

All three 2K/1U rotations are evaluated by five-fold cross-validation. On the unperturbed data, the proposed method attains Accuracy 0.6341±0.0336 and macro-F1 0.5870±0.0618 across the 15 outer-test-fold units. The unperturbed result serves as the baseline for the controlled perturbation analysis. For outer unit *u*, test observation *i*, and feature *j*, the perturbed value is

(17) $$x_{ij}^{\left(\lambda \right)}=x_{ij}+\lambda \sigma_{uj}\epsilon_{ij},\;\epsilon_{ij}∼N\left(0,1\right),$$

where $\sigma_{uj}$ is the sample standard deviation of feature *j* calculated from the selected clean known-class training rows for outer unit *u*. The levels are λ∈{0,0.1,0.2,0.3}. For each nonzero level, three independently seeded noise realizations are evaluated and averaged within each outer fold before the 15-fold mean and sample standard deviation are calculated. Noise is applied exclusively to test observations; the trained triangular models and selected parameters remain fixed.

**Figure 5:**
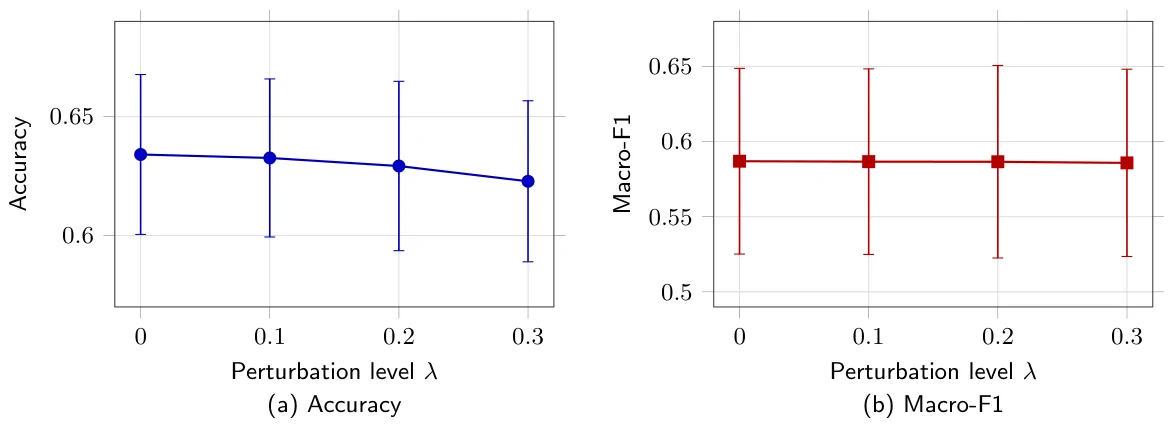
Waveform performance under feature perturbation.

Figure 5 reports mean ± sample standard deviation across the outer-test-fold units. At λ=0.3, Accuracy is 0.6228±0.0338 and macro-F1 is 0.5859±0.0623. Across the investigated range, Accuracy changes by 0.0113 and macro-F1 by only 0.0011, showing that classification performance remains stable on the higher-dimensional noisy evidence as the controlled perturbation increases.

### 5.4. Classification Under Varying Open-World Configurations

Waveform retains the 2K/1U structure used in the preceding open-world evaluations. To examine the method under a different frame composition, the Wall-Following Robot Navigation dataset is further considered. The 24-sensor version contains 5456 sequential observations described by 24 ultrasound readings and four robot-action classes [38]. It supplies real sensor data with unequal class frequencies and supports complete evaluation under both 3K/1U and 2K/2U configurations.

**Figure 6:**
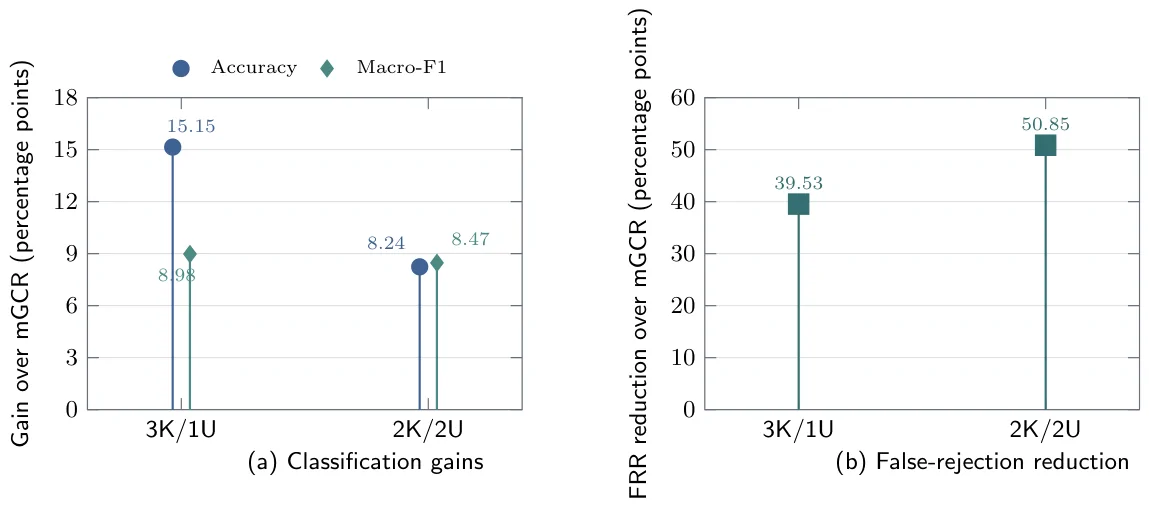
Robot improvements over mGCR.

**Figure 7:**
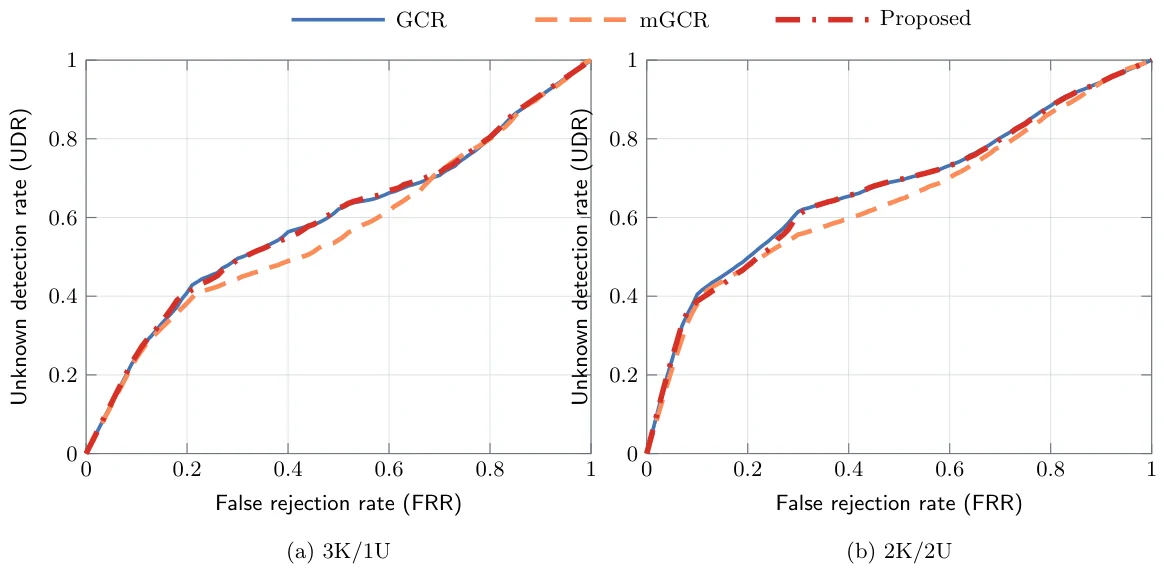
UDR–FRR trade-off curves on the Wall-Following Robot dataset.

Table 11 compares the proposed procedure with GCR and mGCR across all Robot open-world configurations. Relative to mGCR, Accuracy and macro-F1 improve by 15.15 and 8.98 percentage points under 3K/1U, and by 8.24 and 8.47 percentage points under 2K/2U. False rejection is reduced by 39.53 and 50.85 percentage points, respectively. The lower false-rejection rates indicate that the classification gains are achieved while preserving known samples, with the improvement retained when two classes are withheld simultaneously. Figure 6 highlights these performance gains. Figure 7 extends the Robot comparison to the complete UDR–FRR trade-off using the same held-out predictions. The proposed procedure achieves higher configuration-level mean AUROC than mGCR under both 3K/1U (0.5930±0.2147 versus 0.5655±0.2220) and 2K/2U (0.6709±0.2394 versus 0.6468±0.2454), while its overall discrimination remains close to GCR (0.5915±0.2154 and 0.6752±0.2421, respectively). Together with Table 11, these results support the improvement over mGCR both under the original classification rule and across threshold-independent known/unknown discrimination.

### 5.5. Discussion

The principal advantage of the proposed GBBF is that the open-world proposition enters the source-level reference domain before fusion. GBBF-based modification incorporates this proposition into each source while retaining the structure of the observed GBPA. This allows open-world information to participate directly in evidence combination.

The representative Iris and Seeds cases show complementary functions for source modification and combination-rule selection. The GBBF introduces the open-world component, while the combination rule determines how much of that information remains after fusion. In the investigated open-world cases, mGCR retains more open-world support than GCR and allows the unknown class to become dominant when the evidence corresponds to a class outside the current frame.

The low-dimensional experiments make the interaction between source modification and fusion explicit. On Waveform, the proposed classification procedure remains stable in a substantially higher-dimensional noisy setting as controlled feature perturbation increases. The Robot results extend the evaluation to different known/unknown configurations: the gains over mGCR are maintained when one or two classes are treated as unknown. Table 11 reveals distinct known/unknown decision profiles among the fusion strategies. The proposed procedure gives the lowest false-rejection rate in both Robot settings while improving Accuracy and macro-F1 relative to mGCR. It reduces false rejection from 0.5303 to 0.1350 under 3K/1U and from 0.6140 to 0.1055 under 2K/2U. GBBF source modification introduces open-world support where selected, while the conditions based on $K_{\max}$ and $s_{\emptyset }$ route evidence through GCR, mGCR, or GBBF-modified mGCR. The condition-guided routing therefore retains stronger open-world support only when warranted by the current evidence state, preserving more known samples under the original argmax rule. Across the full score range, the proposed procedure has higher mean AUROC than mGCR in both settings and maintains overall discrimination close to GCR.

## 6. Conclusions

This work develops an improved classification method based on a generalized base belief function for incomplete frames of discernment. The proposed function extends the conventional base belief function to the open-world setting by incorporating unknown information into source evidence before fusion. Combined with evidence-condition assessment, GET-compatible fusion, and the subsequent classification decision, it forms a unified procedure for distinguishing known and unknown classes. Numerical examples and the Iris and Seeds experiments provide interpretable evidence of its known-class and open-world behavior. On Waveform, the proposed classification procedure maintains stable classification on higher-dimensional noisy evidence under increasing controlled perturbation. The Robot evaluation shows a selective known/unknown decision with substantially reduced false rejection relative to mGCR and overall discrimination close to GCR across settings with one and two unknown classes. Overall, the results support the proposed GBBF-based classification procedure across noisy evidence and different known/unknown class compositions. Future work will investigate data-adaptive selection of the modification strength and routing parameters, together with sparse focal-set and approximate fusion strategies for larger frames.

## Figures and Tables

**Table 1:** Fusion results for Example 2.

Method	m(a)	m(b)	m(c)	m(a,b)	m(a,c)	m(b,c)	m(a,b,c)	m(∅)
GCR	0.2600	0.2600	0	0	0	0	0	0.4800
mGCR	0.0385	0.0385	0	0	0	0	0	0.9230
mGBPA&GCR	0.2741	0.2521	0.1462	0.0414	0.0414	0.0634	0.0138	0.1676
mGBPA&mGCR	0.1925	0.1770	0.1026	0.0290	0.0290	0.0446	0.0097	0.4156

**Table 2:** Dataset characteristics and evaluation settings.

Dataset	Size (Samples × Attributes)	Classes	Setting	Validation	Characteristics
Iris	150×4	3	Known; 2K/1U	Fixed split	Low-dimensional
Seeds	210×7	3	2K/1U	Fixed split	Different feature distribution
Waveform	5000×21	3	2K/1U	3 rotations × 5 folds	Class overlap; additive noise
Wall-Following Robot	5456×24	4	3K/1U; 2K/2U	4/6 configurations × 5 folds	Sensor data; class imbalance

**Table 3:** Attribute-level GBPAs for the representative Iris known-class instance.

Attribute	m(a)	m(b)	m(c)	m(a,b)	m(a,c)	m(b,c)	m(a,b,c)	m(∅)
SL	0.0006	0	0	0.1873	0	0	0.3124	0.4997
SW	0.5126	0	0.4762	0	0	0	0	0.0112
PL	0.4598	0	0	0	0	0	0	0.5402
PW	0.6557	0	0	0	0	0	0	0.3443

**Table 4:** Fusion results for the representative Iris known-class instance.

Method	m(a)	m(b)	m(c)	m(a,b)	m(a,c)	m(b,c)	m(a,b,c)	m(∅)
GCR	0.9990	0	0	0	0	0	0	0.0010
mGCR	0.9867	0	0	0	0	0	0	0.0133
mGBPA&GCR	0.7770	0.0741	0.1278	0.0076	0.0056	0.0056	0.0006	0.0017
mGBPA&mGCR	0.7640	0.0729	0.1256	0.0075	0.0055	0.0055	0.0006	0.0184

**Table 5:** Original and GBBF-modified GBPAs for the representative Iris unknown-class instance.

		Attributes			
		SL	SW	PL	PW
	Original	0	0	0	0
m(b)	Modified	0.1250	0.1250	0.1250	0.1250
	Original	0	0.1210	0	0
m(c)	Modified	0.1250	0.1855	0.1250	0.1250
	Original	0	0.8000	0	0
m(b,c)	Modified	0.1250	0.5250	0.1250	0.1250
	Original	1	0.0790	1	1
m(∅)	Modified	0.6250	0.1645	0.6250	0.6250

**Table 6:** Fusion results for the representative Iris unknown-class instance.

Method	m(b)	m(c)	m(b,c)	m(∅)
mGBPA&GCR	0.4332	0.4780	0.0486	0.0402
mGBPA&mGCR	0.1512	0.1669	0.0169	0.6650

**Table 7:** Confusion matrix for the Iris open-world test set.

Predicted/Actual	*b*	*c*	∅
*b*	18	1	0
*c*	2	18	0
∅	0	1	50

**Table 8:** Original and GBBF-modified GBPAs for the representative Seeds unknown-class instance.

		Attributes						
Proposition	Item	A	P	C	LK	WK	AC	LKG
m(a)	Original	0.0436	0.1101	0	0.3147	0	0	0.9192
	Modified	0.1468	0.1801	0.1250	0.2824	0.1250	0.1250	0.5846
m(b)	Original	0	0	0.1745	0	0	0.0233	0
	Modified	0.1250	0.1250	0.2123	0.1250	0.1250	0.1367	0.1250
m(a,b)	Original	0	0	0.1706	0	0	0.4604	0
	Modified	0.1250	0.1250	0.2103	0.1250	0.1250	0.3552	0.1250
m(∅)	Original	0.9564	0.8899	0.6549	0.6853	1	0.5163	0.0808
	Modified	0.6032	0.5699	0.4524	0.4676	0.6250	0.3831	0.1654

**Table 9:** Fusion results for the representative Seeds unknown-class instance.

Method	m(a)	m(b)	m(a,b)	m(∅)
mGBPA&GCR	0.8235	0.1717	0.0019	0.0029
mGBPA&mGCR	0.2379	0.0496	0.0005	0.7120

**Table 10:** Confusion matrix for the Seeds open-world test set.

Predicted/Actual	*a*	*b*	∅
*a*	27	3	11
*b*	3	27	0
∅	0	0	59

**Table 11:** Robot classification under varying open-world configurations.

Configuration	Method	Accuracy	Macro-F1	UDR	FRR
3K/1U	GCR	0.5283±0.1611	0.4932±0.1785	0.3812±0.4002	0.1534±0.1218
	mGCR	0.3691±0.1588	0.3940±0.1554	0.5519±0.3283	0.5303±0.1117
	Proposed	0.5206±0.1570	0.4838±0.1788	0.3435±0.3739	0.1350±0.0922
2K/2U	GCR	0.6384±0.2048	0.5969±0.2073	0.4904±0.4246	0.1175±0.1173
	mGCR	0.5189±0.1930	0.4819±0.1729	0.7300±0.2330	0.6140±0.1070
	Proposed	0.6013±0.1962	0.5667±0.1964	0.4348±0.4042	0.1055±0.1142

## Data Availability

The datasets used in this study are publicly available from the UCI Machine Learning Repository [35,36,37,38]. The aggregate results supporting the reported experiments are presented in the article; additional experimental details are available from the authors upon reasonable request.
